# Correlation analysis of epicardial adipose tissue and ventricular myocardial strain in Chinese amateur marathoners using cardiac magnetic resonance

**DOI:** 10.1371/journal.pone.0274533

**Published:** 2022-09-13

**Authors:** Zirong Wang, Tingting Song, Da Yu, Xiaofei Chen, Cailing Pu, Jianping Ding, Xiaoli Ling

**Affiliations:** 1 Department of Radiology, The Affiliated Hospital of Hangzhou Normal University, Hangzhou, Zhejiang, China; 2 Department of Radiology, The Fourth People’s Hospital of Harbin, Harbin, Heilongjiang, China; 3 Department of Ultrasound, The Affiliated Hospital of Hangzhou Normal University, Hangzhou, Zhejiang, China; 4 Department of Radiology, Sir Run Run Shaw Hospital, Zhejiang University School of Medicine, Hangzhou, Zhejiang, China; University of Dundee, UNITED KINGDOM

## Abstract

**Background:**

The volume of epicardial adipose tissue (EAT) is associated with an increased incidence of cardiovascular disease (CVD); however, only a few studies have examined its effect on the myocardial function of endurance in athletes. The association between the EAT and the variation of myocardial function is still unclear in amateur marathoners. Consequently, by using some sedentary individuals as the control, this study aims to evaluate the correlation between the EAT volume and the myocardial strain in the left and right ventricles of Chinese amateur marathoners by cardiac magnetic resonance (CMR).

**Methods:**

A total of 30 amateur marathoners were included as the exercise group and 20 sedentary people as a control group. All participants received the cardiac magnetic resonance (CMR) to measure the left and right ventricular end-diastolic volume, end-systolic volume and volume index, stroke volume and index, cardiac output index, ejection fraction and myocardial mass, the EAT volume, global radial, circumferential, and longi-tudinal strains, and the strain rates of left and right ventricular myocardium.

**Results:**

There was a significant difference in the EAT volume (EATV) index between the exercise group and the control group (26.82±11.76ml/m^2^ vs 37.82±17.15ml/m^2^, P = 0.01). Results from the multivariate linear regression analysis showed that BMI (standardized β = 0.458; P < 0.001) had an independent positive correlation with the EATV index. The EATV index was negatively correlated with the left ventricular global radial strain (GRS) (r = -0.505; P = 0.004) in the exercise group, while it is negatively correlated with right ventricular GRS (r = -0.492; P = 0.027) and positively correlated with global longitudinal strain (GLS) (r = 0.601; P = 0.005) in the control group. In the exercise group, the multivariate linear regression analysis showed that the EATV index (standardized β = -0.429; P = 0.021) was an independent determinant of the left ventricular GRS, and being a male (standardized β = 0.396; P = 0.029) was an independent determinant of the right ventricular GLS.

**Conclusion:**

The EATV index is independently correlated with the left ventricular GRS in the amateur Chinese marathoners, also, the amateur marathon reduces the EATV index and increases the left ventricular myocardial mass, which consequently reduces the adverse effects on myocardial function.

## Introduction

In recent decades, marathon events have been increasing yearly in China; hence, the marathon has become one of the fastest-growing sports attracting an increasing number of participants [1]. Studies have found that as an endurance exercise, a marathon may cause a symmetrical or eccentric remodeling response in the heart, thus, increasing the left and right ventricular cavities by about 3–5% [2], however, the mechanism of ventricular remodeling is still unclear. Recently, people have begun to pay attention to the promotion of the remodeling process by the epicardial adipose tissue (EAT) [3]. EAT is a metabolologically active fat reservoir located between the visceral layer of the pericardium and the myocardial surface and acts as the main source of anti-inflammatory and pro-inflammatory adipokines, as well as an important endocrine and paracrine organ, producing a variety of active substances (such as adiponectin, cytokines and adipokines) that may significantly influence the cardiac function and morphology [4–7]. An animal study has shown that not only does the factors secreted by the EAT in guinea pigs inhibit the contractile function of the myocardial cells, but also, they induce insulin resistance [8]. A genomic epidemiology study on 2471 volunteers from Korea found that the accumulation of epicardial adipose tissue and paracardial adipose tissue was significantly correlated with myocardial remodeling and the subclinical impairment of the left ventricular systolic and diastolic functions [9]. MRI is considered a gold standard in fat measurement, and CMR can easily distinguish between EAT and pericardial fat [10]. Based on the tissue feature tracking technology of the CMR cine sequence, a strain analysis can be performed through the myocardial movement, and changes in the cardiac strain may reflect the changes in the subclinical systolic and diastolic functions in advance [11]. Only a few studies have been conducted on the correlation between the EAT and the myocardial function in amateur marathoners. This study evaluated the quantitative measurement of EATV, the left and right ventricular functions and the myocardial strain capacity of amateur marathoners using CMR quantitative technology and tissue tracking technology to establish the correlation between the EAT and the ventricular myocardial strain.

## Materials and methods

### Study population

The non-randomized controlled study was conducted in accordance with the Helsinki Declaration, and approved by the Ethics Committee of our institution (Approval number: 2021 (E2) -KS-061. Date of ethics approval: 26 February 2021). The purpose, procedures, and risks of the study were communicated to the participants, and written informed consent was obtained from each participant.

**Fig 1:**
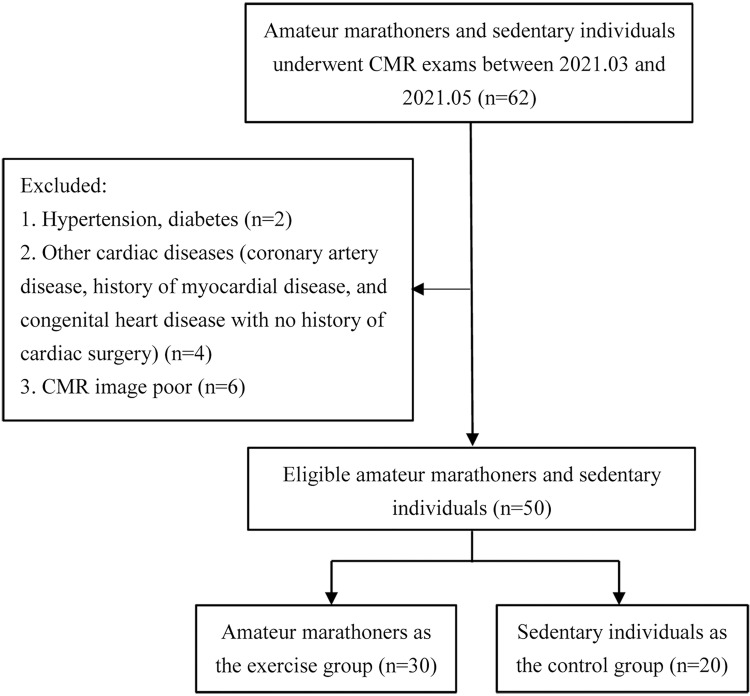
Flow diagram showing the selection and group of Amateur marathoners and sedentary individuals. CMR, cardiac magnetic resonance.

A total of 30 amateur marathoners were recruited and assigned to the exercise group (23 males; age: 31–61 years; average age ± SD: 42.93 years ± 7.24), with 20 sedentary individuals as the control group (14 males; age: 22–43 years; average age ± SD: 34.05 years ±6.33) from March 2021 to May 2021 (Fig 1). Amateur marathon runners were defined as those who had not participated in formal training and whose occupation was not marathon running. Physical training standards: running more than 3 times a week, monthly running volume is more than 50 km. Non-professional marathon runners with more than 1 year of running experience and at least 3 official marathon events were included in this study. The general information of the amateur marathoners, including their name, gender, age, years of running exercise, weekly running distance and marathon times were recorded through a questionnaire survey. Their height, weight, resting heart rate and exercise heart rate were measured. The body surface area (BSA) was calculated based on height and weight using the Mosteller standard equation [12]. The body mass index (BMI) was calculated as the weight divided by the square of the height. The sedentary subjects were defined as those who on average have less than 25 minutes of physical activity per day and have not participated in any marathon [13]. Exclusion criteria: poor or incomplete CMR image, hypertension, diabetes, and coronary artery disease, History of myocardial disease, and Congenital heart disease with no history of cardiac surgery.

### Cardiac MR imaging protocol

CMR images were acquired from all subjects using a 1.5 T MRI with 18-channel phased-array coils (Magnetom Aera, Siemens Healthcare, Germany). A retrospective ECG gated steady-state free precession (SSFP) cine sequence scanning was performed. The subjects were in a supine position during imaging, and held their breath at the end of exhalation to eliminate respiratory motion artifacts, thus, obtaining images of left ventricular short-axis cine sequence and three long-axis cine sequences (two-, three- and four-chamber). The scanning parameters were as follows: layer thickness is 8 mm, interlayer spacing is 2 mm, 48.6 ms repetition time, 1.37 ms echo time, a flip angle of 62°, a FOV of 340 mm×255mm, and a pixel size of 1.8× 1.8 mm.

### Image analysis

**Fig 2:**
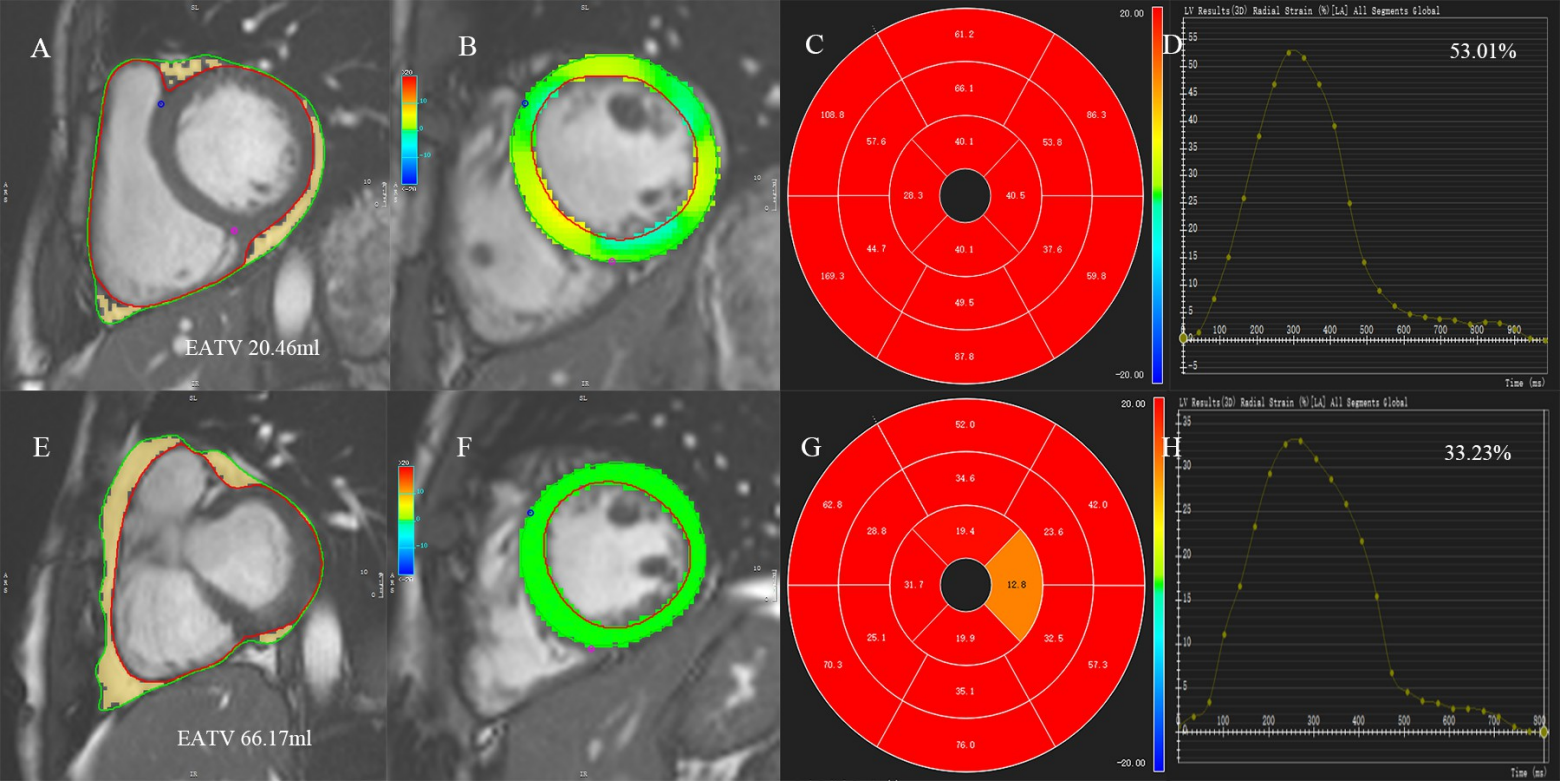
Examples of left ventricular global radial strain (LVGRS) and epicardial adipose tissue volumes (EATV) in 2 Amateur marathoners. A, E: the epicardial and pericardium boundaries were manually delineated at the short axis level of each atrium and the ventricle at the end of diastole, the yellow part between the red and green lines is the epicardial adipose tissue (EAT). B, F: color overlap map of short-axis view. C, G: 16-segment Bull’s eye maps with LVGRS results were displayed. D, F: strain analysis curves of LVGRS. Com-pared with the lower EATV (A), LVGRS was significantly more lower in the Amateur marathoner with larger EATV (E). LVGRS, left ventricular global radial strain; EATV, epicardial adipose tissue volumes; EAT, epicardial adipose tissue.

The analysis of the CMR image was performed using commercially available software (Circle Cardiovascular Imaging 42, Version 5.13.5, Calgary, Canada). The EAT evaluation was performed by standard SSFP cine short-axis sequence, the epicardial and pericardium boundaries were manually delineated at the short axis level of each atrium and the ventricle at the end of diastole, also, the EAT volume was calculated as follows: multiply the sum of the delineated regions of all layers by the thickness of the layer plus the interlayer spacing [14]. The short axis images were imported into the short-axis 3D module of the software, and each part of the endocardial and epicardial boundaries of the left and right ventricles were automatically identified from the bottom to the apex at the end diastole and the systole, with the manual adjustment of some layers. The base of the tricuspid or the mitral valve was the bottom of the right ventricle or the left ventricle [15]. The papillary muscle and the epicardial adipose tissue were excluded. The left and right ventricular functions included the left and right ventricular end-diastolic volumes (EDV), the left and right ventricular end-systolic volumes (ESV), the left and right ventricular end-diastolic volume index (EDVI), the left and right ventricular end-systolic volume index (ESVI), cardiac index (CI), the left and right ventricular ejection fraction (EF), the stroke volume (SV), the stroke volume index (SVI), the left ventricular mass index(LVMI). The left ventricular end-diastole (two-, three-, and four-chamber) and the long axis images were imported into the tissue tracking module to analyze the myocardial strain, which was the myocardium change rate from the end-diastolic initial length (L_I_) to the end-systolic maximum length of the terminal contraction (L_M_), i.e. (L_M_-L_I_)/L_I_×100%. The myocardial strains of the left and right ventricles included the global radial strain (GRS), the global circumcircular strain (GCS), the global longitudinal strain (GLS), the global radial diastolic strain rate (GRSDr), the global circumcircular diastolic strain rate (GCSDr), and the global longitudinal diastolic strain rate (GLSDr), which reflected the myocardial diastolic function (Fig 2).

### Reproducibility

To assess interobserver reproducibility, ten amateur marathon exercise cases and ten healthy controls were randomly selected, and independent image analysis was performed by two radiologists with more than three years of experience. To assess intraobserver reproducibility, the same radiologist re-performed the image analysis on the same 20 subjects after one week.

### Statistical analysis

All continuous data were tested for Gaussian distribution, and the data conforming to the Gaussian distribution were expressed as mean ± SD, however, the non-uniformly distributed data were expressed as median and quartiles (interquartile distance). The differences between the two groups were compared using the independent-samples t-test or the Mann-Whitney U test. Count data were expressed as frequencies (percentages), and the comparisons between two groups were performed using the χ^2^ test or Fisher exact test. The correlation of non-uniformly distributed variables was assessed using Spearman’s rank correlation coefficient. Univariate or multivariate regression analyses were performed to estimate the potential determinants of the association between the EATV index and the longitudinal, radial, and circumferential strains. Inter- and intraobserver repeatability was assessed using the intergroup correlation coefficient (ICC). Two-tailed values of P<0.05 were considered statistically significant differences. All data were analyzed using SPSS (version 23.0; IBM, Armonk, NY, USA) and GraphPad Prism (version 7.00; GraphPad Software Inc., San Diego, CA, USA).

## Results

### General features

Table 1 shows the sporting characteristics of amateur marathon runners. The results of baseline characteristics, EAT, ventricular function and the myocardial strain data for all participants are shown in Table 2. Amateur marathon runners had a smaller EATV index compared to the controls (all P < 0.05). Resting heart rate was lower in amateur marathon runners than in the controls (P < 0.05). LVEDMI, RVEDVI, and the RVESVI were higher in the amateur marathon runners than in the controls (P < 0.05). The left ventricular strain, the overall circumferential strain, the overall circumferential strain rate, and the overall longitudinal strain rate were all lower in the amateur marathon runners than the sedentary controls. The right ventricular strain and the overall circumferential strain rate were lower in the amateur marathon runners than the sedentary controls.

**Table 1:** Description of amateur marathon runner.

Parameter	Amateur marathon Runners (n = 30)
Years of running (y)	5(2–5.25)
Age at start of amateur-running	38.13±7.70
Monthly running distance (km)	200(150–235)
Total running distance (km)	9600(3600–14400)
Number of half-way 21km marathon	5(2–8.25)
Number of 42km marathon races	3(1.5–7.5)

Data are presented as median and interquartile ranges or mean ± SD

**Table 2:** Overall participants’ baseline characteristics and results.

	Amateur marathon runners (n = 30)	Control Group (n = 20)	*P*
Baseline Characteristics			
Age (years)	42.93±7.24	34.05±6.33	<0.001**
Male gender, n (%)	23(76.67)	14(70)	0.60
Heigth (cm)	167.97±7.95	168.75±8.74	0.74
Weight (kg)	62.05±8.95	66.7±14.43	0.21
BMI (kg/m^2^)	21.9±1.64	23.2±3.45	0.13
BSA (m^2^)	1.7±0.16	1.76±0.23	0.28
Heart rate (n/min)	58.67±9.22	68.3±8.84	0.001**
EAT mass (g)	45.26(29.02,58.68)	57.41(37.05,86.29)	0.04*
EATV (ml)	47.53(30.47,62.59)	60.28(38.9,90.61)	0.04*
EATVI (ml/m^2^)	26.82±11.76	37.82±17.15	0.01*
LV Function			
LVEDVI (ml/m^2^)	89.42(78.91,99.81)	83.99(73.92,90.91)	0.06
LVESVI (ml/m^2^)	33.21(28.91,38.93)	28.87(25.91,31)	0.05
LVSVI (ml/m^2^)	55.41(48.19,64.39)	51.23(47.03,58.09)	0.20
LVEF (%)	63.32±9.34	63.75±4.15	0.82
CI (L/min/m^2^)	3.34(2.89,4.1)	3.52(3.33,3.96)	0.15
LVMI (g/m^2^)	57.17(50.64,61.7)	51.21(47.3,53.27)	0.004**
RV Function			
RVEDVI (ml/m^2^)	100.86±15.98	90.98±13.26	0.03*
RVESVI (ml/m^2^)	46.20±8.99	40.67±6.6	0.02*
RVSVI (ml/m^2^)	54.67±10.92	50.74±8.75	0.14
RVEF (%)	54.1±5.58	55.19±4.46	0.47
LV Strain			
LVGRS (%)	37.33±11.53	39.02±9.32	0.59
LVGCS (%)	-19.92±2.89	-21.5±1.85	0.04*
LVGLS (%)	-16.58±1.83	-16.83±1.97	0.64
LVGRSDr (1/s)	-2.23(-2.74,-1.60)	-2.47(-3.43,-2.10)	0.17
LVGCSDr (1/s)	0.92±0.16	1.15±0.21	<0.001**
LVGLSDr (1/s)	0.78±0.16	0.93±0.16	0.002*
RV Strain			
RVGRS (%)	28.74±12.11	30.89±6.74	0.43
RVGCS (%)	-11.83±3.25	-11.22±4.04	0.56
RVGLS (%)	-14.80±2.36	-15.39±3.28	0.46
RVGRSDr (1/s)	-1.58±0.70	-1.82±0.56	0.20
RVGCSDr (1/s)	0.56±0.18	0.80±0.50	0.04*
RVGLSDr (1/s)	0.79±0.18	0.95±0.31	0.05

95% confidence internals are reported in parentheses.

* *p* < 0.05

** *p* < 0.01. BMI, body mass index; BSA, body surface area; EAT, epicardial adipose tissue; EATV, epicardial adipose tissue volume; EATVI, epicardial adipose tissue volume index; LVEDVI, left ventricular end-diastolic volume index; LVESVI, left ventricular end-systolic volume index; LVSVI, left ventricular stroke volume index; LVEF, left ventricular ejection fraction; CI, cardiac index; LVMI, left ventricular mass index; RVEDVI, right ventricular end-diastolic volume index; RVESVI, right ventricular end-systolic volume index; RVSVI, right ventricular stroke volume index; RVEF, right ventricular ejection fraction; LVGRS, left ventricular global radial strain; LVGCS, left ventricular global circumferential strain; LVGLS, left ventricular global longitudinal strain; LVGRSDr, left ventricular global radial strain of diastolic rate; LVGCSDr, left ventricular global circumferential strain of diastolic rate; LVGLSDr, left ventricular global longitudinal strain of diastolic rate; RVGRS, right ventricular global radial strain; RVGCS, right ventricular global circumferential strain; RVGLS, right ventricular global longitudinal strain; RVGRSDr, right ventricular global radial strain of diastolic rate; RVGCSDr, right ventricular global circumferential strain of diastolic rate; RVGLSDr, right ventricular global longitudinal strain of diastolic rate.

### Determinants of the EATV index

Independent determinants of the EATV index were identified by single and multi-factor linear regression analysis, with all significant single factors including age, BMI, heart rate, and amateur marathon exercise (all P < 0.05) included in the multifactor linear regression model. Table 3 shows that only the BMI (standardized β = 0.458; P < 0.001) was independently associated with EATV index.

**Table 3:** Univariable and multivariable linear regression models for EATVI overall (n = 50).

	Univariable		Multivariable	
Variable	Standardized β	*P*	Standardized β	*P*
Age (years)	-0.282	0.047	-0.045	0.756
Male gender (yes or no)	0.225	0.116	-	-
Body mass index (kg/m^2^)	0.514	<0.001	0.458	0.001
Heart rate (n/min)	0.296	0.037	0.192	0.160
LVMI (g/m^2^)	-0.069	0.633	-	-
Amateur marathon runner (yes or no)	-0.362	0.010	-0.156	0.266

LVMI, left ventricular mass index.

### EATV index and the left ventricular strain

**Fig 3:**
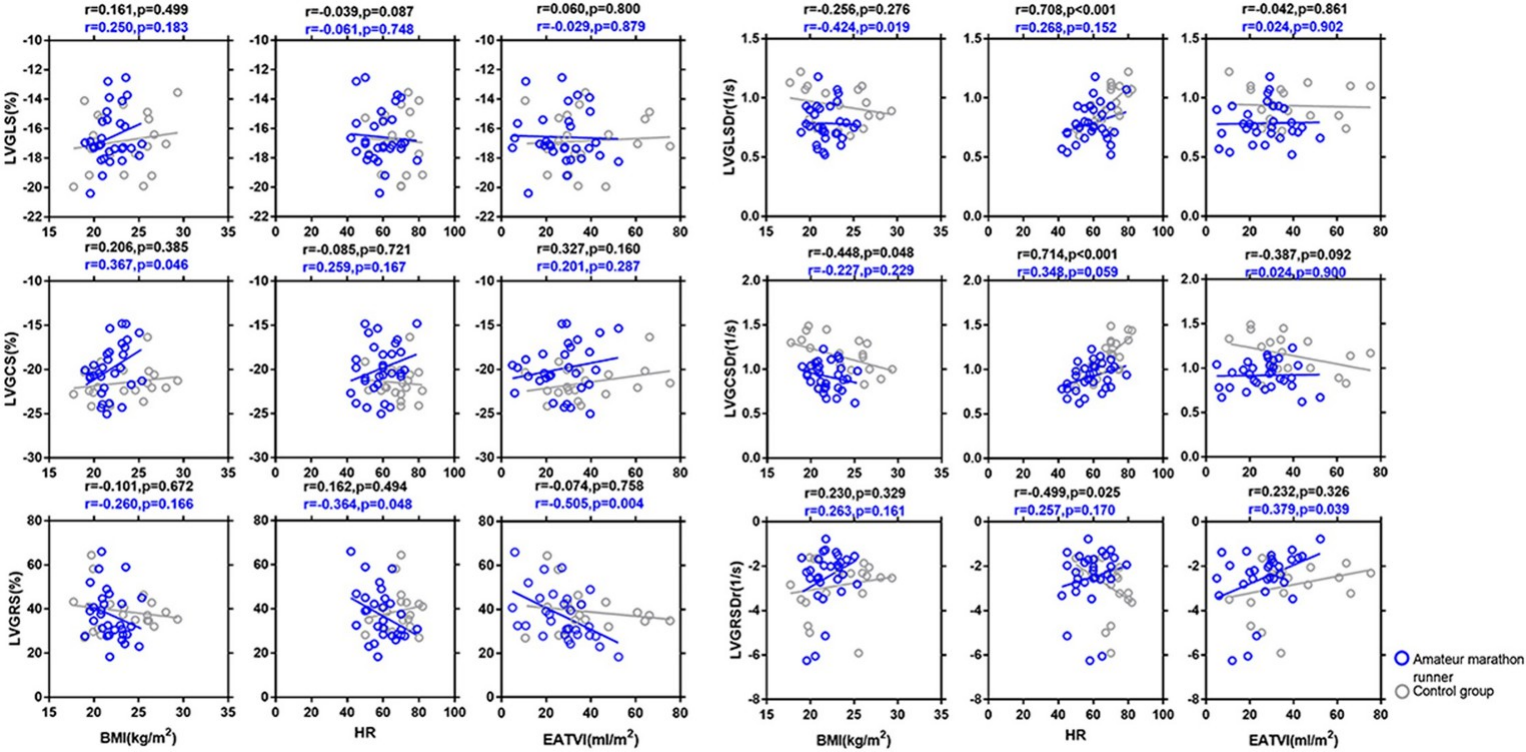
Simple correlations between the cardiac magnetic resonance parameters of left ventricular global radial strain (LVGRS), left ventricular global circumferential strain (LVGCS), left ventricular global longitudinal strain (LVGLS), left ventricular global radial strain of diastolic rate (LVGRSDr), left ventricular global circumferential strain of diastolic rate (LVGCSDr), and left ventricular global longitudinal strain of diastolic rate (LVGLSDr), respectively with body mass index (BMI), heart rate (HR), and epicardial adipose tissue volume index (EATVI) in the amateur marathon runners and control group. A simple regression analysis was made separately in amateur marathon runners (blue circles and lines) and control group (black circles and lines). The R and P-values are shown. LVGRS, left ventricular global radial strain; LVGCS, left ventricular global circumferential strain; LVGLS, left ventricular global longitudinal strain; LVGRSDr, left ventricular global radial strain of diastolic rate; LVGCSDr, left ventricular global circumferential strain of diastolic rate; LVGLSDr, left ventricular global longitudinal strain of diastolic rate; BMI, body mass index; HR, heart rate; EATVI, epicardial adipose tissue volume index.

Fig 3 and S1 Table depict the correlation analysis and the single-factor linear regression analysis of the overall radial, circumferential, and longitudinal left ventricular myocardial strain and the strain rate in amateur marathon runners and the sedentary controls. In the All-volunteer group, the GCS was positively correlated with amateur marathon exercise (r = 0.291; P = 0.04); the GCSDr was positively correlated with heart rate (r = 0.613; P<0.001) and negatively correlated with age, LVEDMI, and amateur marathon exercise (r = -0.380, P = 0.006; r = -0.423, P = 0.002; r = -0.519, P < 0.001); the GLSDr was positively correlated with heart rate (r = 0.548; P < 0.001),and negatively correlated with age and amateur marathon exercise (r = -0.308, P = 0.030; r = -0.409, P = 0.003). In the amateur marathon runners’ group, the GRS was negatively correlated with the EATV index (r = -0.505; P = 0.004, hence, decreasing with increasing EATVI; GCS was positively correlated with BMI, (r = 0.367; P = 0.046) and thus, increasing with increasing BMI. In the sedentary controls, GRSDr was found to be negatively correlated with heart rate (r = -0.499; P = 0.025), decreasing with increasing heart rate; GCSDr was positively correlated with heart rate (r = 0.714; P<0.001) increasing with increasing heart rate and negatively correlated with BMI (r = -0.448; P = 0.048), i.e., decreasing with increasing BMI. GLSDr was positively correlated with heart rate (r = 0.708; P<0.001), and consequently increasing with increasing heart rate.

### EATV index and right ventricular strain

**Fig 4:**
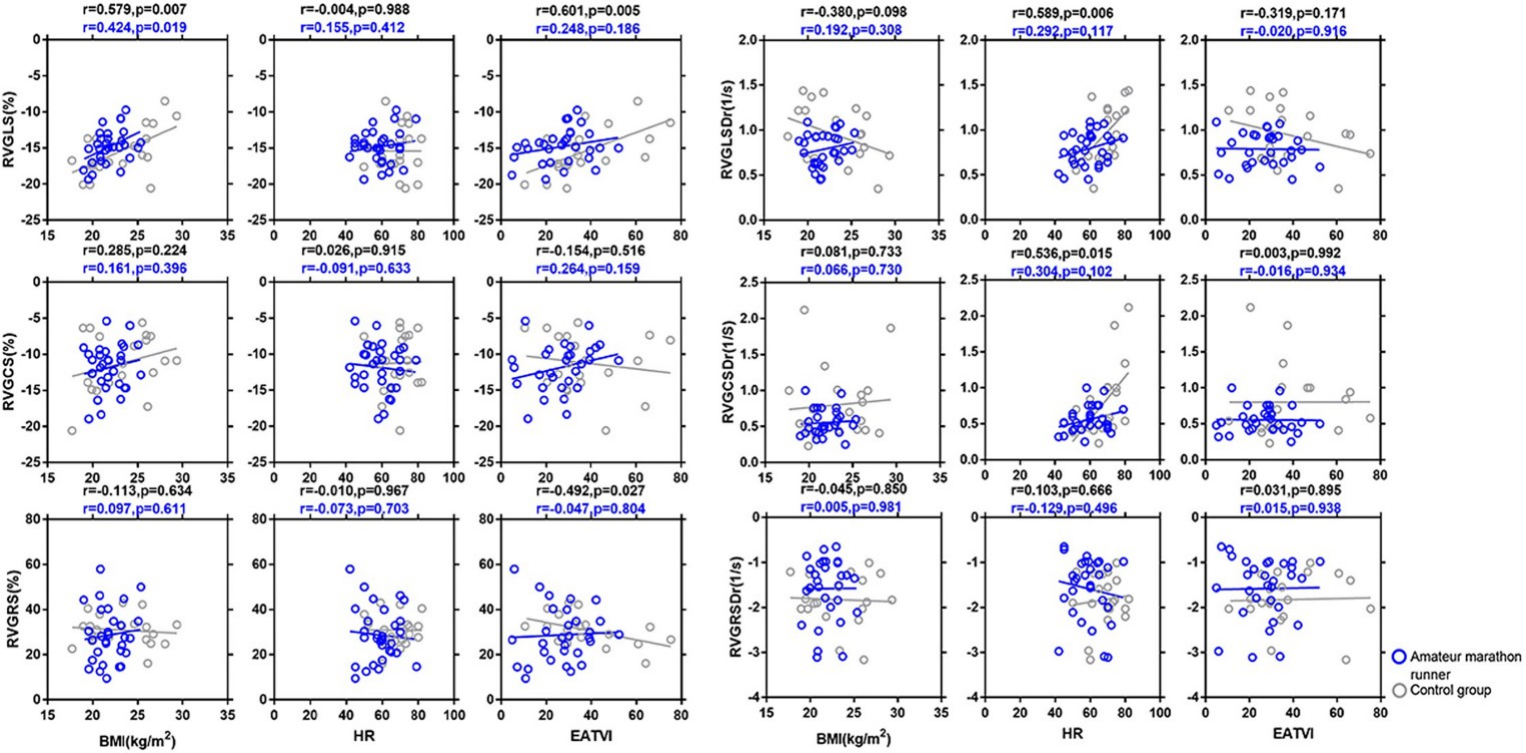
Simple correlations between the cardiac magnetic resonance parameters of right ventricular global radial strain (RVGRS), right ventricular global circumferential strain (RVGCS), right ventricular global longitudinal strain (RVGLS), right ventricular global radial strain of diastolic rate (RVGRSDr), right ventricular global circumferential strain of diastolic rate (RVGCSDr), and right ventricular global longitudinal strain of diastolic rate (RVGLSDr), respectively with body mass index (BMI), heart rate (HR), and epicardial adipose tissue volume index (EATVI) in the amateur marathon runners and control group. A simple regression analysis was made separately in amateur marathon runners (blue circles and lines) and control group (black circles and lines). The R and P-values are shown. RVGRS,right ventricular global radial strain; RVGCS, right ventricular global circumferential strain; RVGLS, right ventricular global longitudinal strain; RVGRSDr, right ventricular global radial strain of diastolic rate; RVGCSDr, right ventricular global circumferential strain of diastolic rate; RVGLSDr, right ventricular global longitudinal strain of diastolic rate; BMI, body mass index; HR, heart rate; EATVI, epicardial adipose tissue volume index.

Fig 4 and S2 Table depict the correlation analysis and the single-factor linear regression analysis of the overall radial, circumferential, and longitudinal right ventricular myocardial strain and the strain rate in amateur marathoners and the sedentary controls. In the all-volunteer group, the GCS was positively correlated with men (r = 0.299; P = 0.035), the GLS was positively correlated with men, BMI, and EATVI (r = 0.344, P = 0.014; r = 0.470, P = 0.001; r = 0.346, P = 0.014); the GCSDr was positively correlated with heart rate (r = 0.490, P< 0.001); GLSDr was positively correlated with heart rate (r = 0.505, P<0.001) and negatively correlated with LVEDMI (r = -0.342, P = 0.015). GLS was positively correlated with males and BMI (r = 0.514, P = 0.004; r = 0.424, P = 0.019), and thus rising with increasing BMI. In the sedentary control group, the GRS was negatively correlated with EATVI (r = -0.492; P = 0.027) decreasing with increasing EATVI, GCS was positively correlated with males and LVEDMI (r = 0.492, P = 0.028; r = 0.453, P = 0.045) increasing with the rise of LVEDMI, also, GLS was positively correlated with BMI, EATVI (r = 0.579, P = 0.007; r = 0.601, P = 0.005), increasing with the rise in BMI and EATVI, furthermore, the GCSDr and heart rate was positively correlated (r = 0.536; P = 0.015), increasing with an increase in the heart rate, and the GLSDr was positively correlated with heart rate (r = 0.589; P = 0.006), increasing with the rise in the heart rate.

### Determinants of myocardial strain in the left ventricle

Table 4 and S3 Table demonstrate the LV myocardial strain and the determinants of the strain rate as revealed by the univariate and multivariate linear regression analyses. In the amateur marathon exercise group, the GRS univariate linear regression analysis was included first because the EATV index had a moderate negative association with GRS (r = -0.505; P = 0.004), and the variables with P<0.05, including heart rate and EATV index, were included in the multivariate linear regression analysis in a forced entry mode. The EATV index (standardized β = -0.429; P = 0.021) was found to be an independent determinant of GRS in the multiple linear regression model. Only BMI (standardized β = 0.367; P = 0.046) was statistically significant in the GCS univariate linear regression analysis.

**Table 4:** Univariable and multivariable linear regression models for LV myocardial strain amateur marathon runners (n = 30).

	Global radial strain (%)				Global circumferential strain (%)				Global longitudinal strain (%)			
	Univariable		Multivariable		Univariable		Multivariable		Univariable		Multivariable	
Variable	Standardized β	P	Standardized β	P	Standardized β	P	Standardized β	P	Standardized β	P	Standardized β	P
Age	0.071	0.711	-	-	-0.077	0.686	-	-	-0.213	0.259	-	-
Male gender	-0.065	0.733	-	-	0.290	0.120	-	-	0.196	0.300	-	-
BMI	-0.260	0.166	-	-	0.367	0.046	-	-	0.250	0.183	-	-
Heart rate	-0.364	0.048	-0.205	0.250	0.246	0.189	-	-	-0.054	0.776	-	-
LVMI	0.197	0.297	-	-	0.099	0.602	-	-	0.088	0.467	-	-
EATVI	-0.505	0.004	-0.429	0.021	0.088	0.645	-	-	-0.024	0.902	-	-

BMI, body mass index; LVMI, left ventricular mass index; EATVI, epicardial adipose tissue volume index.

### Determinants of myocardial strain in the right ventricle

Table 5 and S4 Table demonstrate the right ventricular myocardial strain and the determinants of the strain rate screened by the univariate and multifactor linear regression analyses. In the amateur marathoner group, the variables with P<0.05, including the male gender, BMI, and EATVI were included in the multivariate linear regression analysis in a forced entry mode in the GLS univariate linear regression analysis. Male (standardized β = 0.396; P = 0.029) was an independent determinant of the GLS in the multiple linear regression model.

**Table 5:** Univariable and multivariable linear regression models for RV myocardial strain amateur marathon runners (n = 30).

	Global radial strain (%)				Global circumferential strain (%)				Global longitudinal strain (%)			
	Univariable		Multivariable		Univariable		Multivariable		Univariable		Multivariable	
Variable	Standardized β	P	Standardized β	P	Standardized β	P	Standardized β	P	Standardized β	P	Standardized β	P
Age	0.052	0.785	-	-	-0.014	0.941	-	-	0.171	0.365	-	-
Male gender	-0.063	0.741	-	-	0.202	0.283	-	-	0.525	0.003	0.396	0.029
BMI	0.097	0.611	-	-	0.161	0.396	-	-	0.424	0.019	0.228	0.196
Heart rate	-0.073	0.703	-	-	-0.093	0.626	-	-	0.156	0.411	-	-
LVMI	0.105	0.580	-	-	-0.007	0.973	-	-	0.228	0.226	-	-
EATVI	-0.008	0.968	-	-	0.256	0.171	-	-	0.323	0.082	-	-

BMI, body mass index; LVMI, left ventricular mass index; EATVI, epicardial adipose tissue volume index.

### Intraobserver and interobserver reproducibility

The results showed good reproducibility of the intraobserver and interobserver EATV index and all strain measurements. S5 Table shows the ICC values for intra-observer and interobserver reproducibility.

## Discussion

This study assessed the correlation between EATV and LV&RV myocardial strain in Chinese amateur marathon runners. The following are the strengths and novelties of this paper: (1) The LV and RV myocardial function was quantified using CMR and tissue tracking techniques. (2) The EAT volumes were quantified using the CMR gold standard rather than cardiac CT volume measurements or the echocardiographic local thickness measurements. (3) The EATV index was significantly lower in the amateur marathon exercise group than in the sedentary control group. (4) BMI was independently associated with the EATV index. (5) The multiple regression analysis showed that the EATV index was independently associated with a progressive lower overall radial strain in amateur marathon runners.

There was a significant difference in the EAT volume (EATV) index between the exercise group and the control group (26.82±11.76ml/m^2^ vs 37.82±17.15ml/m^2^, P = 0.01). Amateur marathon runners have a smaller EATV index, and amateur marathon exercises can reduce the EATV index and this is consistent with the findings from previous studies where ultramarathoners had a smaller EATV [16]. Results from the multivariate linear regression analysis showed that BMI (standardized β = 0.458; P < 0.001) had an independent positive correlation with the EATV index. BMI was independently associated with the EATV index, and an increase in the BMI translates into a gradual increment in the EATV index, which is consistent with the findings of Ng, Song, and others [17, 18]. Resting heart rate was reduced in amateur marathon runners. Also, LVMI, RVEDVI, and RVESVI were higher in amateur marathon runners than in the controls, and this is also consistent with the athletes’ cardiac performance of ventricular wall thickening and chamber dilation [19] and similar to the results of Lewicka et al. [20, 21]. Firstly, long-term endurance exercise changes the thickness of the left ventricular wall and the left ventricular myocardial mass. In addition, long-term endurance exercise training induces cardiomyocyte hypertrophy, which increases the formation of the myocardial cross-bridge and myogenic fibers, resulting in an increased left ventricular myocardial mass [22]. In the present study, the overall left ventricular circumferential strain, the overall circumferential strain rate, the overall longitudinal strain rate, and the overall right ventricular circumferential strain rate were found to be lower in amateur marathoners than in the sedentary controls, and this is consistent with studies on ultramarathoners in which the overall left ventricular (radial, circumferential, and longitudinal) the strain and strain rate were found to be lower when compared with healthy controls [23] and were within normal values [24].

EAT is deposited in the fat layer between the pericardium and the myocardium, covering about 80% of the myocardium and accounting for 20% of the heart’s mass [25]. There is a close functional and anatomical relationship between the EAT and the myocardium. Both have the same microcirculation, adipose tissue and the myocardium are in close contact without fascia between them, therefore, there is paracrine mediation between the adipose tissue and the myocardium [26]. EAT is derived from the visceral pleural mesoderm, and the pericardial fat is derived from the primitive thoracic mesenchyme [7]. EAT is composed of fat cells, ganglia, nerves, and inflammatory factors, stromovascular and immune cells. The effects of EAT on the heart include mechanical action, myocardial energy source, and the synthesis, production, and secretion of biologically active molecules [25–28].

EAT is the main source of free and abundant fatty acids and numerous biologically active molecules (such as adiponectin, leptin, and inflammatory cytokines). EAT can either be protective or harmful for coronary arteries and cardiomyocytes depending on the changes in the EATV and the microenvironment [26, 29]. As an active endocrine organ, EAT directly transports fatty acids to the myocardium and functions as an energy source for the myocardium [30]. However, the excessive secretion of large amounts of fatty acids by EAT exceeds the oxidative capacity of the myocardial, and excessive fatty acids produce the accumulation of ceramide and other intermediates, and this subsequently results in the apoptosis and fibrosis of the myocardial cell [31, 32]. The mate analysis by Gonzalo et al. showed that physical exercise could significantly reduce EAT, and the effect was still significant after the removal of the research having a greater impact on the results after sensitivity analysis (Bairapareddy et al. [33]) [34]. In a randomized clinical trial, the EAT quality of 50 obese patients was significantly reduced by 32% and 24% after 12 weeks of intervention of endurance or resistance training, this is an indication that different exercise methods have potential preventive significance in the reduction of heart fat in obese patients [35]. This study showed that the EAT volume index of the amateur marathon group was significantly lower than that of the control group, which coincided with the cited research results.

An increase in EATV increases the secretion of pro-inflammatory adipocytokines (leptin, mucin, interleukin 1-β, interleukin-6, and anti-bodies). The direct paracrine action leads to an increase in inflammation and fibrosis of the adjacent myocardium [5, 36]. Vitro studies in rat cardiomyocytes have found that increased expression of miR-208a secreted by EAT could damage mitochondrial β-oxidation, thereby changing the contractile function of the myocardium [37] through the renin-angiotensin-aldosterone system. Tran et al. discovered that the increase of miR-155 and miR-302a-3p in the plasma of patients with atrial fibrillation was positively correlated with the thickness of EAT, which may be related to heart remodeling [38, 39]. An increase in the volume of TNF-α secreted by EAT could induce changes in cardiomyocyte apoptosis, cardiac dilatation, and extracellular matrix remodeling [40]. The increase in EATV could cause the accumulation of triglycerides in the myocardium. Studies by Malavazos et al. indicated an independent correlation between the EAT thickness of echocardiography and the accumulation of triglycerides in the myocardium of the magnetic resonance spectroscopy and the subclinical myocardial dysfunction in diabetic patients [41]. The NG et al. research team found that increased EATV index and insulin resistance were independently related to the accumulation of myocardial fat and myocardial interstitial fibrosis, with a decrease in the overall left ventricular myocardial strain [42]. The vitro studies of EAT on the biopsy of patients with type 2 diabetes revealed that the adipokines (activin A, angiopoietin-2, and CD14) secreted by the EAT lowered the Ca^2+^ flux of cardiomyocytes and the expression of sarcoplasmic endoplasmic reticulum ATPase 2a, and reduced cardiomyocytes shortening of the sarcomere [43]. This study found that in the amateur marathon group, the EATV index was negatively correlated with the overall radial strain of the myocardium (r = -0.505; P = 0.004), which is consistent with the findings of Ng ACT et al. [17]. Previous studies have shown that the left ventricular GRS was positively correlated with the LV mass index [44], and the left ventricular GRS was higher in hypertensive patients with left ventricular hypertrophy [45]. This study found that the EATV index had nothing to do with the LV mass index. This may be an indication that the left ventricular GRS of amateur marathon runners was independently affected by the EATV index, which subsequently affected the left ventricular myocardial function and structural changes. As the EATV index increased, the left ventricular GRS gradually decreased. It was found in the right ventricular myocardial strain study that the result that being male was an independent determinant of right ventricular GLS in the multiple linear regression model was identical with the results from a previous study by Truong et al. [46]. In the control group, the EATV index was positively correlated with the right ventricular GLS, and negatively correlated with the right ventricular GRS. EATV was remarkably higher than the amateur marathon group and more distributed on the right ventricular sidewall, which may indicate the secretion of more EAT inflammation molecules in the right ventricular sidewall. The increase in sex factors, adipokines, TNF-α, and myocardial fat accumulation was more likely to affect the right ventricular myocardial function, also, muscle fat accumulation was more likely to affect the right ventricular myocardial function, and these findings are consistent with findings from a previous report [47]. Furthermore, myocardial contraction can be classified based on the myocardium involved. GLS: the subendocardial myocardial fiber contraction led to longitudinal shortening, expressed as a negative value, GCS: the subepicardial myocardial fiber contraction led to circumferential shortening, expressed as a negative value, and GRS: the shrinkage of the transmembrane fibers led to radial thickening, which was expressed as a positive value [11]. CMR imaging has high spatial and temporal resolution and can provide information on cardiac morphology, function, hemodynamics and tissue characteristics in a single examination without exposing the patient to ionizing radiation. CMR feature tracking technology is an excellent non-invasive tool based on conventional CMR cine images, simple, practical, easy to obtain and less time consuming than other techniques. It enables quantitative assessment of myocardial compliance based on whole heart and segmental myocardial function analysis and provides detailed data on cardiac systole and diastole, providing useful information for early detection and intervention of heart disease. CMR can be used as a gold standard for assessing whole body adipose tissue and has been widely used and is now considered the simplest non-invasive method for measuring EAT volume and mass. The EAT can be visualized as a high-signal, light gray area located between the low-signal myocardium and the visceral pericardium. Measurement of EAT through CMR does not require the use of gadolinium contrast agents, the safety of which has become increasingly controversial in the last few years.

This study is a single-center cross-sectional study with a relatively small number of only Chinese participants, therefore the applicability of the results and findings to other ethnic groups require further relevant research. Secondly, this study found that the EATV index was an independent determinant of CMR left ventricular GRS, and this did not necessarily indicate that the increase in EATV index was the cause of the left ventricular myocardial dysfunction that will eventually lead to heart failure. Instantly, the vitro studies of EAT on the biopsy of diabetic patients showed that the adipokines secreted by EAT reduced the metabolism of Ca^2+^ in cardiomyocytes and decreased the shortening of the cardiomyocyte sarcomere [43]. Thirdly, previous studies have shown that a variety of factors secreted by EAT were related to myocardial function [36, 40]. However, volunteers enrolled in this study were all healthy, and the EAT tissue biopsy could not be obtained for confirmation purposes.

## Conclusions

This study revealed that the EATV index of amateur marathoners is independently related to the left ventricular GRS, and amateur marathon exercise reduces EATV, and this translates into the reduction of the adverse effects on myocardial function. This finding may help to further understand the extent of amateur marathon exercise and the relation-ship between EATV and myocardial contraction as well as the diastolic function.

## Supporting information

S1 Checklist STROBE statement—checklist of items that should be included in reports of observational studies. (DOCX)

S1 Table Correlation analysis between variables and LV myocardial strain. (DOCX)

S2 Table Correlation analysis between variables and RV myocardial strain. (DOCX)

S3 Table Univariable and multivariable linear regression models for LV myocardial strain amateur marathoners. (DOCX)

S4 Table Univariable and multivariable linear regression models for RV myocardial strain amateur marathoners. (DOCX)

S5 Table Intra-observer and inter-observer measurement variabilities for EATV index and myocardial strain. (DOCX)

## References

[1] Zuo Y, Zou L, Zhang M, Smith L, Yang L, Loprinzi PD, et al. The Temporal and Spatial Evolution of Marathons in China from 2010 to 2018. Int J Environ Res Public Health. 2019;16(24):5046. doi: 10.3390/ijerph16245046 . 31835745 PMC6950243

[2] D’Silva A, Bhuva AN, van Zalen J, Bastiaenen R, Abdel-Gadir A, Jones S, et al. Cardiovascular Remodeling Experienced by Real-World, Unsupervised, Young Novice Marathon Runners. Frontiers in Physiology. 2020;11(232). doi: 10.3389/fphys.2020.00232 32256389 PMC7093496

[3] Parisi V, Cabaro S, D’Esposito V, Petraglia L, Conte M, Campana P, et al. Epicardial Adipose Tissue and IL-13 Response to Myocardial Injury Drives Left Ventricular Remodeling After ST Elevation Myocardial Infarction. Frontiers in Physiology. 2020;11(1306). doi: 10.3389/fphys.2020.575181 33178043 PMC7593695

[4] Salazar J, Luzardo E, Mejías JC, Rojas J, Ferreira A, Rivas-Ríos JR, et al. Epicardial Fat: Physiological, Pathological, and Therapeutic Implications. Cardiology Research and Practice. 2016;2016:1291537. doi: 10.1155/2016/1291537 27213076 PMC4861775

[5] Packer M. Epicardial Adipose Tissue May Mediate Deleterious Effects of Obesity and Inflammation on the Myocardium. Journal of the American College of Cardiology. 2018;71(20):2360–72. doi: 10.1016/j.jacc.2018.03.509 29773163

[6] Iacobellis G, Pistilli D, Gucciardo M, Leonetti F, Miraldi F, Brancaccio G, et al. Adiponectin expression in human epicardial adipose tissue in vivo is lower in patients with coronary artery disease. Cytokine. 2005;29(6):251–5. doi: 10.1016/j.cyto.2004.11.002 15749025

[7] Sacks HS, Fain JN. Human epicardial adipose tissue: A review. American Heart Journal. 2007;153(6):907–17. doi: 10.1016/j.ahj.2007.03.019 17540190

[8] Greulich S, de Wiza DH, Preilowski S, Ding Z, Mueller H, Langin D, et al. Secretory products of guinea pig epicardial fat induce insulin resistance and impair primary adult rat cardiomyocyte function. Journal of Cellular and Molecular Medicine. 2011;15(11):2399–410. doi: 10.1111/j.1582-4934.2010.01232.x 21143387 PMC3822951

[9] Kim J-S, Kim SW, Lee JS, Lee SK, Abbott R, Lee KY, et al. Association of pericardial adipose tissue with left ventricular structure and function: a region‐specific effect? Cardiovascular Diabetology. 2021;20(1):26. doi: 10.1186/s12933-021-01219-4 33494780 PMC7836147

[10] Kessels K, Cramer MJM, Velthuis B. Epicardial adipose tissue imaged by magnetic resonance imaging: an important risk marker of cardiovascular disease. Heart. 2006;92(7):962. doi: 10.1136/hrt.2005.074872 16775103 PMC1860684

[11] Claus P, Omar AMS, Pedrizzetti G, Sengupta PP, Nagel E. Tissue Tracking Technology for Assessing Cardiac Mechanics: Principles, Normal Values, and Clinical Applications. JACC: Cardiovascular Imaging. 2015;8(12):1444–60. doi: 10.1016/j.jcmg.2015.11.001 26699113

[12] Wood AM, Hoffmann KR, Lipton MJ. Cardiac function. Quantification with magnetic resonance and computed tomography. Radiol Clin North Am. 1994;32(3):553–79. . 8184029

[13] Roeh A, Lembeck M, Papazova I, Pross B, Hansbauer M, Schoenfeld J, et al. Marathon running improves mood and negative affect. Journal of Psychiatric Research. 2020;130:254–9. doi: 10.1016/j.jpsychires.2020.08.005 32854076

[14] Parisi V, Petraglia L, Formisano R, Caruso A, Grimaldi MG, Bruzzese D, et al. Validation of the echocardiographic assessment of epicardial adipose tissue thickness at the Rindfleisch fold for the prediction of coronary artery disease. Nutrition, Metabolism and Cardiovascular Diseases. 2020;30(1):99–105. doi: 10.1016/j.numecd.2019.08.007 31648886

[15] Scharf M, Brem MH, Wilhelm M, Schoepf UJ, Uder M, Lell MM. Atrial and Ventricular Functional and Structural Adaptations of the Heart in Elite Triathletes Assessed with Cardiac MR Imaging. Radiology. 2010;257(1):71–9. doi: 10.1148/radiol.10092377 20807850

[16] Konwerski M, Postuła M, Barczuk-Falęcka M, Czajkowska A, Mróz A, Witek K, et al. Epicardial Adipose Tissue and Cardiovascular Risk Assessment in Ultra-Marathon Runners: A Pilot Study. Int J Environ Res Public Health. 2021;18(6). doi: 10.3390/ijerph18063136 33803664 PMC8002849

[17] Ng ACT, Goo SY, Roche N, Van Der Geest RJ, Wang WYS. Epicardial Adipose Tissue Volume and Left Ventricular Myocardial Function Using 3-Dimensional Speckle Tracking Echocardiography. Canadian Journal of Cardiology. 2016;32(12):1485–92. doi: 10.1016/j.cjca.2016.06.009 27720272

[18] Song DK, Hong YS, Lee H, Oh J-Y, Sung Y-A, Kim Y. Increased Epicardial Adipose Tissue Thickness in Type 2 Diabetes Mellitus and Obesity. Diabetes & Metabolism Journal. 2015;39(5):405. doi: 10.4093/dmj.2015.39.5.405 26566498 PMC4641970

[19] Crouse SF, White S, Erwin JP, Meade TH, Martin SE, Oliver JM, et al. Echocardiographic and Blood Pressure Characteristics of First-Year Collegiate American-Style Football Players. American Journal of Cardiology. 2016;117(1):131–4. doi: 10.1016/j.amjcard.2015.09.049 26554673

[20] Lewicka-Potocka Z, Dąbrowska-Kugacka A, Lewicka E, Kaleta AM, Dorniak K, Daniłowicz-Szymanowicz L, et al. The "athlete’s heart" features in amateur male marathon runners. Cardiology journal. 2021;28(5):707–15. Epub 01/07. doi: 10.5603/CJ.a2019.0110 . 31909474 PMC8428944

[21] D’Ascenzi F, Anselmi F, Piu P, Fiorentini C, Carbone SF, Volterrani L, et al. Cardiac Magnetic Resonance Normal Reference Values of Biventricular Size and Function in Male Athlete’s Heart. JACC: Cardiovascular Imaging. 2019;12(9):1755–65. doi: 10.1016/j.jcmg.2018.09.021 30553678

[22] Kim YJ, Park KM. Effects of Super-ultramarathon Running on Cardiac Structure and Function in Middle-aged Men. Journal of cardiovascular imaging. 2020;28(3):202–10. doi: 10.4250/jcvi.2020.0020 . 32583637 PMC7316553

[23] Małek ŁA, Mazurkiewicz Ł, Marszałek M, Barczuk-Falęcka M, Simon JE, Grzybowski J, et al. Deformation Parameters of the Heart in Endurance Athletes and in Patients with Dilated Cardiomyopathy-A Cardiac Magnetic Resonance Study. Diagnostics (Basel). 2021;11(2):374. doi: 10.3390/diagnostics11020374 . 33671723 PMC7926616

[24] Taylor RJ, Moody WE, Umar F, Edwards NC, Taylor TJ, Stegemann B, et al. Myocardial strain measurement with feature-tracking cardiovascular magnetic resonance: normal values. European Heart Journal–Cardiovascular Imaging. 2015;16(8):871–81. doi: 10.1093/ehjci/jev006 25711353

[25] Iacobellis G, Bianco AC. Epicardial adipose tissue: emerging physiological, pathophysiological and clinical features. Trends Endocrinol Metab. 2011;22(11):450–7. Epub 08/16. doi: 10.1016/j.tem.2011.07.003 . 21852149 PMC4978122

[26] Iacobellis G, Corradi D, Sharma AM. Epicardial adipose tissue: anatomic, biomolecular and clinical relationships with the heart. Nature Clinical Practice Cardiovascular Medicine. 2005;2(10):536–43. doi: 10.1038/ncpcardio0319 16186852

[27] Iacobellis G. Epicardial and Pericardial Fat: Close, but Very Different. Obesity. 2009;17(4):625–. doi: 10.1038/oby.2008.575 19322142

[28] Villasante Fricke AC, Iacobellis G. Epicardial Adipose Tissue: Clinical Biomarker of Cardio-Metabolic Risk. Int J Mol Sci. 2019;20(23):5989. doi: 10.3390/ijms20235989 . 31795098 PMC6929015

[29] Mazurek T, Zhang L, Zalewski A, Mannion JD, Diehl JT, Arafat H, et al. Human Epicardial Adipose Tissue Is a Source of Inflammatory Mediators. Circulation. 2003;108(20):2460–6. doi: 10.1161/01.CIR.0000099542.57313.C5 14581396

[30] KankaanpäÄ M, Lehto H-R, PäRkkä JP, Komu M, Viljanen A, Ferrannini E, et al. Myocardial Triglyceride Content and Epicardial Fat Mass in Human Obesity: Relationship to Left Ventricular Function and Serum Free Fatty Acid Levels. The Journal of Clinical Endocrinology & Metabolism. 2006;91(11):4689–95. doi: 10.1210/jc.2006-0584 16926257

[31] Zhou Y-T, Grayburn P, Karim A, Shimabukuro M, Higa M, Baetens D, et al. Lipotoxic heart disease in obese rats: Implications for human obesity. Proceedings of the National Academy of Sciences. 2000;97(4):1784. doi: 10.1073/pnas.97.4.1784 10677535 PMC26513

[32] McGavock JM, Victor RG, Unger RH, Szczepaniak LS. Adiposity of the Heart*, Revisited. Annals of Internal Medicine. 2006;144(7):517–24. doi: 10.7326/0003-4819-144-7-200604040-00011 16585666

[33] B KC, Maiya A, R PK, Nayak K, Guddattu V, Nayak V. Effect of aerobic exercise on echocardiographic epicardial adipose tissue thickness in overweight individuals. Diabetes, Metabolic Syndrome and Obesity: Targets and Therapy. 2018;Volume 11:303–12. doi: 10.2147/dmso.s145862 29950876 PMC6018891

[34] Saco‐Ledo G, Valenzuela PL, Castillo‐García A, Arenas J, León‐Sanz M, Ruilope LM, et al. Physical exercise and epicardial adipose tissue: A systematic review and meta‐analysis of randomized controlled trials. Obesity Reviews. 2021;22(1). doi: 10.1111/obr.13103 32692478

[35] Christensen RH, Wedell-Neergaard A-S, Lehrskov LL, Legaard GE, Dorph E, Larsen MK, et al. Effect of Aerobic and Resistance Exercise on Cardiac Adipose Tissues. JAMA Cardiology. 2019;4(8):778. doi: 10.1001/jamacardio.2019.2074 31268469 PMC6613292

[36] Cherian S, Lopaschuk GD, Carvalho E. Cellular cross-talk between epicardial adipose tissue and myocardium in relation to the pathogenesis of cardiovascular disease. AMERICAN JOURNAL OF PHYSIOLOGY-ENDOCRINOLOGY AND METABOLISM. 2012;303(8):E937–E49. doi: 10.1152/ajpendo.00061.2012 WOS:000309982000001. 22895783

[37] Blumensatt M, Fahlbusch P, Hilgers R, Bekaert M, Herzfeld De Wiza D, Akhyari P, et al. Secretory products from epicardial adipose tissue from patients with type 2 diabetes impair mitochondrial β-oxidation in cardiomyocytes via activation of the cardiac renin–angiotensin system and induction of miR-208a. Basic Research in Cardiology. 2017;112(1). doi: 10.1007/s00395-016-0591-0 27864612

[38] Khanh-Van T, Majka O, Sanghai S, Sardana M, Lessard D, Milstone Z, et al. Micro-RNAs Are Related to Epicardial Adipose Tissue in Participants With Atrial Fibrillation: Data From the MiRhythm Study. FRONTIERS IN CARDIOVASCULAR MEDICINE. 2019;6. doi: 10.3389/fcvm.2019.00115 WOS:000480758300001. 31475159 PMC6702296

[39] Santos D, Carvalho E. Adipose-related microRNAs as modulators of the cardiovascular system: the role of epicardial adipose tissue. JOURNAL OF PHYSIOLOGY-LONDON. 2021. doi: 10.1113/JP280917 WOS:000696338600001. 34455587

[40] Patel VB, Shah S, Verma S, Oudit GY. Epicardial adipose tissue as a metabolic transducer: role in heart failure and coronary artery disease. Heart Failure Reviews. 2017;22(6):889–902. doi: 10.1007/s10741-017-9644-1 28762019

[41] Malavazos AE, Di Leo G, Secchi F, Lupo EN, Dogliotti G, Coman C, et al. Relation of Echocardiographic Epicardial Fat Thickness and Myocardial Fat. The American Journal of Cardiology. 2010;105(12):1831–5. doi: 10.1016/j.amjcard.2010.01.368 20538139

[42] Ng ACT, Strudwick M, Van Der Geest RJ, Ng ACC, Gillinder L, Goo SY, et al. Impact of Epicardial Adipose Tissue, Left Ventricular Myocardial Fat Content, and Interstitial Fibrosis on Myocardial Contractile Function. Circulation: Cardiovascular Imaging. 2018;11(8). doi: 10.1161/circimaging.117.007372 30354491

[43] Greulich S, Maxhera B, Vandenplas G, De Wiza DH, Smiris K, Mueller H, et al. Secretory Products From Epicardial Adipose Tissue of Patients With Type 2 Diabetes Mellitus Induce Cardiomyocyte Dysfunction. Circulation. 2012;126(19):2324–34. doi: 10.1161/CIRCULATIONAHA.111.039586 23065384

[44] Haggerty CM, Jing L, Fornwalt BK. Of mice (dogs) and men: getting to the heart of obesity-associated cardiac dysfunction. Diabetologia. 2016;59(1):9–12. doi: 10.1007/s00125-015-3798-y 26518683 PMC4764985

[45] Kouzu H, Yuda S, Muranaka A, Doi T, Yamamoto H, Shimoshige S, et al. Left Ventricular Hypertrophy Causes Different Changes in Longitudinal, Radial, and Circumferential Mechanics in Patients with Hypertension: A Two-Dimensional Speckle Tracking Study. JOURNAL OF THE AMERICAN SOCIETY OF ECHOCARDIOGRAPHY. 2011;24(2):192–9. doi: 10.1016/j.echo.2010.10.020 WOS:000286809300011. 21145703

[46] Truong VT, Safdar KS, Kalra DK, Gao X, Ambach S, Taylor MD, et al. Cardiac magnetic resonance tissue tracking in right ventricle: Feasibility and normal values. Magnetic Resonance Imaging. 2017;38:189–95. doi: 10.1016/j.mri.2017.01.007 28093270

[47] Gokdeniz T, Erkol A, Kalaycioglu E, Aykan AC, Gul I, Boyaci F, et al. Relation of Epicardial Fat Thickness to Subclinical Right Ventricular Dysfunction Assessed by Strain and Strain Rate Imaging in Subjects with Metabolic Syndrome: A Two-Dimensional Speckle Tracking Echocardiography Study. ECHOCARDIOGRAPHY-A JOURNAL OF CARDIOVASCULAR ULTRASOUND AND ALLIED TECHNIQUES. 2015;32(2):248–56. doi: 10.1111/echo.12635 WOS:000349676600007. 24815416

