# Supplementary material for: Correlation analysis of epicardial adipose tissue and ventricular myocardial strain in Chinese amateur marathoners using cardiac magnetic resonance
Source: PLoS One. 2022 Sep 13;17(9):e0274533. doi: 10.1371/journal.pone.0274533 (PMC9470000; doi:10.1371/journal.pone.0274533)
Supplement: S1 Checklist — (DOCX) [file pone.0274533.s001.docx]

STROBE Statement—checklist of items that should be included in reports of observational studies

|  | Item No. | Recommendation | Page  No. | Relevant text from manuscript |
| --- | --- | --- | --- | --- |
| **Title and abstract** | 1 | (*a*) Indicate the study’s design with a commonly used term in the title or the abstract | Page 1, line 1. | Correlation analysis of epicardial adipose tissue and ventricular myocardial strain in Chinese amateur marathoners using cardiac magnetic resonance |
|  |  | (*b*) Provide in the abstract an informative and balanced summary of what was done and what was found | Page 1, line 36. | Methods: All participants received the cardiac magnetic resonance (CMR) to measure the EAT volume, global radial, circumferential, and longi-tudinal strains, and the strain rates of left and right ventricular myocardium.  Conclusion: The EATV index is independently correlated with the left ventricular GRS in the amateur Chinese marathoners, also, the amateur marathon reduces the EATV index and increases the left ventricular myocardial mass, which consequently reduces the adverse effects on myocardial function. |
| Introduction | | | |  |
| Background/rationale | 2 | Explain the scientific background and rationale for the investigation being reported | Page 2,line 60. | Studies have found that as an endurance exercise, a marathon may cause a symmetrical or eccentric remodeling response in the heart, thus, increasing the left and right ventricular cavities by about 3-5%, however, the mechanism of ventricular remodeling is still unclear. |
| Objectives | 3 | State specific objectives, including any prespecified hypotheses | Page 2,line 81. | This study evaluated the quantitative measurement of EATV, the left and right ventricular functions and the myocardial strain capacity of amateur marathoners using CMR quantitative technology and tissue tracking technology to establish the correlation between the EAT and the ventricular myocardial strain. |
| Methods | | | |  |
| Study design | 4 | Present key elements of study design early in the paper | Page 2,line 88. | The non-randomized controlled study was conducted in accord-ance |
| Setting | 5 | Describe the setting, locations, and relevant dates, including periods of recruitment, exposure, follow-up, and data collection | Page 3, line 92. | A total of 30 amateur marathoners were recruited and assigned to the exercise group. |
| Participants | 6 | (*a*) *Cohort study*—Give the eligibility criteria, and the sources and methods of selection of participants. Describe methods of follow-up  *Case-control study*—Give the eligibility criteria, and the sources and methods of case ascertainment and control selection. Give the rationale for the choice of cases and controls  *Cross-sectional study*—Give the eligibility criteria, and the sources and methods of selection of participants | Pag e3,line 95. | Amateur marathoners in this study refer to non-professional marathoners who have not undergone any routine physical training, including marathoners who have been engaged in running exercise for more than 1 year |
|  |  | (*b*) *Cohort study*—For matched studies, give matching criteria and number of exposed and unexposed  *Case-control study*—For matched studies, give matching criteria and the number of controls per case |  |  |
| Variables | 7 | Clearly define all outcomes, exposures, predictors, potential confounders, and effect modifiers. Give diagnostic criteria, if applicable | Page 4,line 150. | The EAT evaluation was performed by standard SSFP cine short-axis sequence |
| Data sources/ measurement | 8* | For each variable of interest, give sources of data and details of methods of assessment (measurement). Describe comparability of assessment methods if there is more than one group | *Page 4,line 153.* | *The EAT volume was calculated as follows: multiply the sum of the delineated regions of all layers by the thickness of the layer plus the interlayer spacing [14].* |
| Bias | 9 | Describe any efforts to address potential sources of bias | Page 12,line 432. | Inter- and Intra-observer Reproducibility |
| Study size | 10 | Explain how the study size was arrived at | Page 3,line 92. | A total of 30 amateur marathoners were recruited and assigned to the exercise group (23 females; age: 31-61 years; average age ± SD: 42.93 years ± 7.24), with 20 sedentary individuals as the control group (14 males; age: 22-43 years; average age ± SD: 34.05 years ±6.33) from March 2021 to May 2021. |

Continued on next page

| Quantitative variables | 11 | Explain how quantitative variables were handled in the analyses. If applicable, describe which groupings were chosen and why | Page 4, line 165. | The left ventricular end-diastole (two-, three-, and four-chamber) and the long axis images were imported into the tissue tracking module to analyze the myocardial strain, which was the myocardium change rate from the end-diastolic initial length (L_I_) to the end-systolic maximum length of the terminal contraction (L_M_), i.e. (L_M_-L_I_)/L_I_×100% |
| --- | --- | --- | --- | --- |
| Statistical methods | 12 | (*a*) Describe all statistical methods, including those used to control for confounding | Page 5,line217 | All continuous data were tested for Gaussian distribution. |
|  |  | (*b*) Describe any methods used to examine subgroups and interactions | Page 5,line220 | The differences between the two groups were compared using the independent-samples t-test or the Mann-Whitney U test. |
|  |  | (*c*) Explain how missing data were addressed |  |  |
|  |  | (*d*) *Cohort study*—If applicable, explain how loss to follow-up was addressed  *Case-control study*—If applicable, explain how matching of cases and controls was addressed  *Cross-sectional study*—If applicable, describe analytical methods taking account of sampling strategy | Page 5,line224 | Univariate or multivariate regression analyses were per-formed to estimate the potential determinants of the association between the EATV index and the longitudinal, radial, and circumferential strains |
|  |  | (*e*) Describe any sensitivity analyses | Page 5,line227 | Two-tailed values of P<0.05 were considered statistically significant differences |
| Results | | | | |
| Participants | 13* | (a) Report numbers of individuals at each stage of study—eg numbers potentially eligible, examined for eligibility, confirmed eligible, included in the study, completing follow-up, and analysed | Page 3, line 92. | A total of 30 amateur marathoners were recruited and assigned to the exercise group, with 20 sedentary individuals as the control group. |
|  |  | (b) Give reasons for non-participation at each stage | Page 3, line 106. | Exclusion criteria |
|  |  | (c) Consider use of a flow diagram | Page 3, line 134. | Fig1. Flow diagram showing the selection and group of Amateur marathoners and sedentary individuals. |
| Descriptive data | 14* | (a) Give characteristics of study participants (eg demographic, clinical, social) and information on exposures and potential confounders | Page 6, line 233. | General Features |
|  |  | (b) Indicate number of participants with missing data for each variable of interest |  |  |
|  |  | (c) *Cohort study*—Summarise follow-up time (eg, average and total amount) | Page 6, line 256. | Table 2 |
| Outcome data | 15* | *Cohort study*—Report numbers of outcome events or summary measures over time | *Page 7, line 274.* | *Determinants of the EATV index* |
|  |  | *Case-control study—*Report numbers in each exposure category, or summary measures of exposure |  |  |
|  |  | *Cross-sectional study—*Report numbers of outcome events or summary measures |  |  |
| Main results | 16 | (*a*) Give unadjusted estimates and, if applicable, confounder-adjusted estimates and their precision (eg, 95% confidence interval). Make clear which confounders were adjusted for and why they were included | Page 10, line 403. | Determinants of myocardial strain in the left ventricle |
|  |  | (*b*) Report category boundaries when continuous variables were categorized |  |  |
|  |  | (*c*) If relevant, consider translating estimates of relative risk into absolute risk for a meaningful time period | Page 8, line 284. | EATV index and the left ventricular strain |

Continued on next page

| Other analyses | 17 | Report other analyses done—eg analyses of subgroups and interactions, and sensitivity analyses |  |  |
| --- | --- | --- | --- | --- |
| Discussion | | | | |
| Key results | 18 | Summarise key results with reference to study objectives | Page 11, line 438 | This study assessed the correlation between EATV and LV&RV myocardial strain in Chinese amateur marathon runners. |
| Limitations | 19 | Discuss limitations of the study, taking into account sources of potential bias or imprecision. Discuss both direction and magnitude of any potential bias | Page 14, line 544 | This study is a single-center study |
| Interpretation | 20 | Give a cautious overall interpretation of results considering objectives, limitations, multiplicity of analyses, results from similar studies, and other relevant evidence | Page 14, line 557 | Conclusions |
| Generalisability | 21 | Discuss the generalisability (external validity) of the study results | Page 14, line 561 | This finding may help to further understand the extent of amateur marathon exercise and the relation-ship between EATV and myocardial contraction as well as the diastolic function. |
| Other information | |  | | |
| Funding | 22 | Give the source of funding and the role of the funders for the present study and, if applicable, for the original study on which the present article is based | Page 15, line 568 | The Basic Public Welfare Research Pro-ject of Zhejiang Province |

*Give information separately for cases and controls in case-control studies and, if applicable, for exposed and unexposed groups in cohort and cross-sectional studies.

**Note:** An Explanation and Elaboration article discusses each checklist item and gives methodological background and published examples of transparent reporting. The STROBE checklist is best used in conjunction with this article (freely available on the Web sites of PLoS Medicine at http://www.plosmedicine.org/, Annals of Internal Medicine at http://www.annals.org/, and Epidemiology at http://www.epidem.com/). Information on the STROBE Initiative is available at www.strobe-statement.org.
