# Supplementary material for: Correlation analysis of epicardial adipose tissue and ventricular myocardial strain in Chinese amateur marathoners using cardiac magnetic resonance
Source: PLoS One. 2022 Sep 13;17(9):e0274533. doi: 10.1371/journal.pone.0274533 (PMC9470000; doi:10.1371/journal.pone.0274533)
Supplement: S1 Table — (DOCX) [file pone.0274533.s002.docx]

| **S1 Table Correlation analysis between variables and LV myocardial strain**  A Overall（n=50） | | | | | | | | | | |  | | | | |
| --- | --- | --- | --- | --- | --- | --- | --- | --- | --- | --- | --- | --- | --- | --- | --- |
|  | LVGRS (%) | | LVGCS (%) | | LVGLS (%) | | LVGRSDr (1/S) | | LVGCSDr (1/S) | | | LVGLSDr (1/S) | |  |  |
| Variable | r | P | r | P | r | P | r | P | r | P | | r | P | |  |
| Age（years） | 0.030 | 0.839 | 0.150 | 0.299 | -0.055 | 0.702 | 0.083 | 0.566 | -0.380 | 0.006 | | -0.308 | 0.030 | |  |
| Male gender（yes or no） | -0.093 | 0.520 | 0.169 | 0.241 | 0.092 | 0.527 | 0.003 | 0.983 | -0.161 | 0.263 | | -0.123 | 0.394 | |  |
| Body mass index（kg/m^2^） | -0.129 | 0.372 | 0.177 | 0.218 | 0.122 | 0.400 | 0.175 | 0.225 | -0.171 | 0.235 | | -0.069 | 0.636 | |  |
| Heart rate (n/min) | -0.106 | 0.464 | -0.006 | 0.967 | -0.078 | 0.592 | -0.131 | 0.363 | 0.631 | ＜0.001 | | 0.548 | ＜0.001 | |  |
| LVMI (g/m^2^) | -0.052 | 0.721 | 0.180 | 0.212 | 0.060 | 0.678 | 0.050 | 0.732 | -0.423 | 0.002 | | -0.241 | 0.092 | |  |
| Amateur marathon runner（yes or no） | -0.109 | 0.451 | 0.291 | 0.040 | 0.017 | 0.907 | 0.197 | 0.171 | -0.519 | ＜0.001 | | -0.409 | 0.003 | |  |
| EATVI (ml/m^2^) | -0.274 | 0.054 | -0.005 | 0872 | -0.023 | 0.873 | 0.246 | 0.085 | 0.084 | 0.560 | | 0.062 | 0.667 | |  |
| Abbreviations: LVGRS, left ventricular global radial strain; LVGCS, left ventricular global circumferential strain; LVGLS, left ventricular global longitudinal strain; LVGRSDr, left ventricular global radial strain of diastolic rate; LVGCSDr, left ventricular global circumferential strain of diastolic rate; LVGLSDr, left ventricular global longitudinal strain of diastolic rate; LVMI, left ventricular mass index; EATVI, epicardial adipose tissue volume index. | | | | | | | | | | | | | | |  |

| B Amateur marathon runner（n=30） | | | | | | | | | | | | |
| --- | --- | --- | --- | --- | --- | --- | --- | --- | --- | --- | --- | --- |
|  | LVGRS (%) | | LVGCS (%) | | LVGLS (%) | | LVGRSDr (1/S) | | LVGCSDr (1/S) | | LVGLSDr (1/S) | |
| Variable | r | P | r | P | r | P | r | P | r | P | r | P |
| Age（years） | 0.071 | 0.711 | -0.077 | 0.686 | -0.213 | 0.259 | -0.127 | 0.503 | -0.198 | 0.295 | -0.009 | 0.963 |
| Male gender（yes or no） | -0.178 | 0.348 | 0.269 | 0.151 | 0.123 | 0.518 | -0.073 | 0.703 | -0.287 | 0.124 | -0.210 | 0.266 |
| Body mass index（kg/m2） | -0.260 | 0.166 | 0.367 | 0.046 | 0.250 | 0.183 | 0.263 | 0.161 | -0.227 | 0.229 | -0.022 | 0.907 |
| Heart rate (n/min) | -0.364 | 0.048 | 0.259 | 0.167 | -0.061 | 0.748 | 0.257 | 0.170 | 0.348 | 0.059 | 0.268 | 0.152 |
| LVMI (g/m2) | 0.197 | 0.297 | 0.099 | 0.602 | 0.088 | 0.644 | -0.135 | 0.475 | -0.182 | 0.336 | -0.135 | 0.477 |
| Amateur marathon runner（yes or no） |  |  |  |  |  |  |  |  |  |  |  |  |
| EATVI (ml/m2) | -0.505 | 0.004 | 0.201 | 0.287 | -0.029 | 0.879 | 0.379 | 0.039 | 0.024 | 0.900 | 0.024 | 0.902 |
| Abbreviations: See Supplementary Table S1 A. | | | | | | | | | | | | |

| C Control group（n=20） | | | | | | | | | | | | | | | | | | | | | | | |
| --- | --- | --- | --- | --- | --- | --- | --- | --- | --- | --- | --- | --- | --- | --- | --- | --- | --- | --- | --- | --- | --- | --- | --- |
|  | LVGRS (%) | | | LVGCS (%) | | | | LVGLS (%) | | | | LVGRSDr (1/S) | | | | LVGCSDr (1/S) | | | | LVGLSDr (1/S) | | | |
| Variable | r | P | | r | | P | | r | | P | | r | | P | | r | | P | | r | | P | |
| Age（years） | -0.060 | 0.802 | | 0.157 | | 0.509 | | 0.056 | | 0.814 | | 0.172 | | 0.469 | | -0.023 | | 0.922 | | 0.056 | | 0.814 | |
| Male gender（yes or no） | 0.076 | 0.751 | | -0.003 | | 0.991 | | 0.100 | | 0.674 | | -0.083 | | 0.727 | | 0.020 | | 0.933 | | 0.123 | | 0.606 | |
| Body mass index（kg/m2） | -0.101 | 0.672 | | 0.206 | | 0.385 | | 0.161 | | 0.499 | | 0.230 | | 0.329 | | -0.448 | | 0.048 | | -0.256 | | 0.276 | |
| Heart rate (n/min) | 0.162 | 0.494 | | -0.085 | | 0.721 | | -0.039 | | 0.087 | | -0.499 | | 0.025 | | 0.714 | | ＜0.001 | | 0.708 | | ＜0.001 | |
| LVMI (g/m2) | -0.200 | 0.398 | | 0.164 | | 0.490 | | 0.259 | | 0.271 | | 0.035 | | 0.885 | | -0.310 | | 0.183 | | -0.025 | | 0.917 | |
| Amateur marathon runner（yes or no） |  |  |  | |  | |  | |  | |  | |  | |  | |  | |  | |  | |  |
| EATVI (ml/m2) | -0.074 | 0.758 | | 0.327 | | 0.160 | | 0.060 | | 0.800 | | 0.232 | | 0.326 | | -0.387 | | 0.092 | | -0.042 | | 0.861 | |
| Abbreviations: See Supplementary Table S1 A. | | | | | | | | | | | | | | | | | | | | | | | |
