# Supplementary material for: Correlation analysis of epicardial adipose tissue and ventricular myocardial strain in Chinese amateur marathoners using cardiac magnetic resonance
Source: PLoS One. 2022 Sep 13;17(9):e0274533. doi: 10.1371/journal.pone.0274533 (PMC9470000; doi:10.1371/journal.pone.0274533)
Supplement: S2 Table — (DOCX) [file pone.0274533.s003.docx]

| **S2 Table Correlation analysis between variables and RV myocardial strain**  A Overall（n=50） | | | | | | | | | |  | | | |
| --- | --- | --- | --- | --- | --- | --- | --- | --- | --- | --- | --- | --- | --- |
|  | RVGRS (%) | | RVGCS (%) | | RVGLS (%) | | RVGRSDr (1/S) | | RVGCSDr (1/S) | | RVGLSDr (1/S) | |  |
| Variable | r | P | r | P | r | P | r | P | r | P | r | P |  |
| Age（years） | -0.020 | 0.888 | -0.136 | 0.347 | 0.118 | 0.415 | 0.124 | 0.390 | -0.156 | 0.279 | -0.197 | 0.171 |  |
| Male gender（yes or no） | -0.052 | 0.719 | 0.299 | 0.035 | 0.344 | 0.014 | 0.052 | 0.719 | -0.022 | 0.879 | -0.163 | 0.259 |  |
| Body mass index（kg/m2） | 0.030 | 0.835 | 0.245 | 0.087 | 0.470 | 0.001 | -0.065 | 0.656 | 0.160 | 0.266 | -0.112 | 0.437 |  |
| Heart rate (n/min) | 0.001 | 0.989 | 0.005 | 0.974 | 0.021 | 0.887 | -0.131 | 0.365 | 0.490 | ＜0.001 | 0.505 | ＜0.001 |  |
| LVMI (g/m2) | -0.043 | 0.766 | 0.125 | 0.385 | 0.241 | 0.091 | 0.203 | 0.158 | -0.187 | 0.194 | -0.342 | 0.015 |  |
| Amateur marathon runner（yes or no） | -0.156 | 0.281 | -0.079 | 0.584 | 0.103 | 0.475 | 0.242 | 0.090 | -0.228 | 0.111 | -0.265 | 0.063 |  |
| EATVI (ml/m2) | -0.006 | 0.967 | 0.115 | 0.425 | 0.346 | 0.014 | -0.022 | 0.879 | 0.175 | 0.225 | -0.013 | 0.930 |  |
| Abbreviations: RVGRS, right ventricular global radial strain; RVGCS, right ventricular global circumferential strain; RVGLS, right ventricular global longitudinal strain; RVGRSDr, right ventricular global radial strain of diastolic rate; RVGCSDr, right ventricular global circumferential strain of diastolic rate; RVGLSDr, right ventricular global longitudinal strain of diastolic rate; LVMI, left ventricular mass index; EATVI, epicardial adipose tissue volume index. | | | | | | | | | | | | |  |

| B Amateur marathon runner（n=30） | | | | | | | | | | | | |
| --- | --- | --- | --- | --- | --- | --- | --- | --- | --- | --- | --- | --- |
|  | RVGRS (%) | | RVGCS (%) | | RVGLS (%) | | RVGRSDr (1/S) | | RVGCSDr (1/S) | | RVGLSDr (1/S) | |
| Variable | r | P | r | P | r | P | r | P | r | P | r | P |
| Age（years） | 0.052 | 0.785 | -0.014 | 0.941 | 0.171 | 0.365 | -0.157 | 0.409 | -0.151 | 0.426 | -0.215 | 0.253 |
| Male gender（yes or no） | -0.105 | 0.582 | 0.150 | 0.428 | 0.514 | 0.004 | 0.223 | 0.236 | -0.087 | 0.649 | -0.360 | 0.051 |
| Body mass index（kg/m2） | 0.097 | 0.611 | 0.161 | 0.396 | 0.424 | 0.019 | 0.005 | 0.981 | 0.066 | 0.730 | 0.192 | 0.308 |
| Heart rate (n/min) | -0.073 | 0.703 | -0.091 | 0.633 | 0.155 | 0.412 | -0.129 | 0.496 | 0.304 | 0.102 | 0.292 | 0.117 |
| LVMI (g/m2) | 0.105 | 0.580 | 0.007 | 0.973 | 0.228 | 0.226 | 0.128 | 0.501 | -0.051 | 0.790 | -0.120 | 0.529 |
| Amateur marathon runner（yes or no） |  |  |  |  |  |  |  |  |  |  |  |  |
| EATVI (ml/m2) | 0.047 | 0.804 | 0.264 | 0.159 | 0.248 | 0.186 | 0.015 | 0.938 | -0.016 | 0.934 | -0.020 | 0.916 |
| Abbreviations: See Supplementary Table S2 A. | | | | | | | | | | | | |

| C Control group（n=20） | | | | | | | | | | | | | |  |
| --- | --- | --- | --- | --- | --- | --- | --- | --- | --- | --- | --- | --- | --- | --- |
|  | RVGRS (%) | | RVGCS (%) | | RVGLS (%) | | RVGRSDr (1/S) | | | RVGCSDr (1/S) | | RVGLSDr (1/S) | |  |
| Variable | r | P | r | P | r | P | | r | P | r | P | R | P |  |
| Age（years） | 0.222 | 0.347 | -0.243 | 0.302 | -0.048 | 0.840 | | 0.434 | 0.056 | 0.163 | 0.492 | 0.147 | 0.535 |  |
| Male gender（yes or no） | 0.057 | 0.812 | 0.492 | 0.028 | 0.133 | 0.578 | | -0.256 | 0.276 | -0.022 | 0.926 | 0.057 | 0.812 |  |
| Body mass index（kg/m2） | -0.113 | 0.634 | 0.285 | 0.224 | 0.579 | 0.007 | | -0.045 | 0.850 | 0.081 | 0.733 | -0.380 | 0.098 |  |
| Heart rate (n/min) | -0.010 | 0.967 | 0.026 | 0.915 | -0.004 | 0.988 | | 0.103 | 0.666 | 0.536 | 0.015 | 0.589 | 0.006 |  |
| LVMI (g/m2) | 0.338 | 0.144 | 0.453 | 0.045 | 0.117 | 0.622 | | -0.180 | 0.448 | -0.156 | 0.512 | -0.368 | 0.110 |  |
| Amateur marathon runner（yes or no） |  |  |  |  |  |  | |  |  |  |  |  |  |  |
| EATVI (ml/m2) | -0.492 | 0.027 | -0.154 | 0.516 | 0.601 | 0.005 | | 0.031 | 0.895 | 0.003 | 0.992 | -0.319 | 0.171 |  |
| Abbreviations: See Supplementary Table S2 A. | | | | | | | | | | | | | | |
