# Supplementary material for: Correlation analysis of epicardial adipose tissue and ventricular myocardial strain in Chinese amateur marathoners using cardiac magnetic resonance
Source: PLoS One. 2022 Sep 13;17(9):e0274533. doi: 10.1371/journal.pone.0274533 (PMC9470000; doi:10.1371/journal.pone.0274533)
Supplement: S3 Table — (DOCX) [file pone.0274533.s004.docx]

| **S3 Table Univariable and multivariable linear regression models for LV myocardial strain amateur marathoners (n=30)** | | | | | | | | | | | | |  |
| --- | --- | --- | --- | --- | --- | --- | --- | --- | --- | --- | --- | --- | --- |
|  | Global radial strain of diastolic rate（1/s） | | | | Global circumferential strain of diastolic rate（1/s） | | | | Global longitudinal strain of diastolic rate（1/s） | | | | |
|  | Univariable |  | Multivariable |  | Univariable |  | Multivariable |  | Univariable |  | Multivariable |  | |
| Variable | Standardized β | P | Standardized β | P | Standardized β | P | Standardized β | P | Standardized β | P | Standardized β | P | |
| Age | 0.038 | 0.841 | -0.023 | 0.906 | -0.198 | 0.295 | -0.264 | 0.166 | -0.009 | 0.963 | -0.071 | 0.732 | |
| Male gender | -0.073 | 0.703 | -0.108 | 0.666 | -0.277 | 0.138 | -0.434 | 0.084 | -0.221 | 0.240 | -0.316 | 0.250 | |
| BMI | 0.291 | 0.119 | 0.462 | 0.051 | -0.227 | 0.229 | -0.212 | 0.341 | -0.022 | 0.907 | 0.042 | 0.864 | |
| Heart rate | 0.168 | 0.374 | -0.015 | 0.943 | 0.359 | 0.051 | 0.365 | 0.088 | 0.273 | 0.144 | 0.260 | 0.266 | |
| LVMI | -0.137 | 0.471 | -0.333 | 0.240 | -0.182 | 0.336 | 0.339 | 0.221 | -0.135 | 0.477 | 0.132 | 0.664 | |
| EATVI | 0.312 | 0.094 | 0.242 | 0.251 | 0.083 | 0.661 | 0.097 | 0.634 | 0.099 | 0.602 | 0.066 | 0.770 | |
| Abbreviations: BMI, body mass index; LVMI, left ventricular mass index; EATVI, epicardial adipose tissue volume index. | | | | | | | | | | | | |  |
