# Supplementary material for: Correlation analysis of epicardial adipose tissue and ventricular myocardial strain in Chinese amateur marathoners using cardiac magnetic resonance
Source: PLoS One. 2022 Sep 13;17(9):e0274533. doi: 10.1371/journal.pone.0274533 (PMC9470000; doi:10.1371/journal.pone.0274533)
Supplement: S4 Table — (DOCX) [file pone.0274533.s005.docx]

| **S4 Table Univariable and multivariable linear regression models for RV myocardial strain amateur marathoners (n=30)** | | | | | | | | | | | | |
| --- | --- | --- | --- | --- | --- | --- | --- | --- | --- | --- | --- | --- |
|  | Global radial strain of diastolic rate（1/s） | | | | Global circumferential strain of diastolic rate（1/s） | | | | Global longitudinal strain of diastolic rate（1/s） | | | |
|  | Univariable |  | Multivariable |  | Univariable |  | Multivariable |  | Univariable |  | Multivariable |  |
| Variable | Standardized β | P | Standardized β | P | Standardized β | P | Standardized β | P | Standardized β | P | Standardized β | P |
| Age | -0.157 | 0.409 | -0.042 | 0.527 | -0.151 | 0.426 | -0.172 | 0.400 | -0.215 | 0.253 | -0.140 | 0.425 |
| Male gender | 0.167 | 0.378 | 0.303 | 0.715 | -0.069 | 0.718 | -0.140 | 0.597 | -0.345 | 0.062 | -0.223 | 0.203 |
| BMI | 0.005 | 0.981 | -0.209 | 0.742 | 0.066 | 0.730 | 0.153 | 0.526 | 0.192 | 0.308 | 0.156 | 0.374 |
| Heart rate | -0.135 | 0.476 | -0.189 | 0.577 | 0.306 | 0.100 | 0.396 | 0.089 | 0.300 | 0.108 | 0.327 | 0.082 |
| LVMI | 0.128 | 0.501 | -0.171 | 0.843 | -0.051 | 0.790 | 0.104 | 0.726 | -0.120 | 0.529 | 0.096 | 0.611 |
| EATVI | 0.012 | 0.950 | 0.364 | 0.805 | -0.038 | 0.844 | -0.180 | 0.418 | -0.007 | 0.971 | -0.108 | 0.567 |
| Abbreviations: BMI, body mass index; LVMI, left ventricular mass index; EATVI, epicardial adipose tissue volume index. | | | | | | | | | | | | |
