# Supplementary material for: Correlation analysis of epicardial adipose tissue and ventricular myocardial strain in Chinese amateur marathoners using cardiac magnetic resonance
Source: PLoS One. 2022 Sep 13;17(9):e0274533. doi: 10.1371/journal.pone.0274533 (PMC9470000; doi:10.1371/journal.pone.0274533)
Supplement: S5 Table — (DOCX) [file pone.0274533.s006.docx]

**S5 Table Intra-observer and inter-observer measurement variabilities for EATVI and myocardial strain**

| Variable | Intra-observer | Inter-observer |
| --- | --- | --- |
|  | ICC (95% CI) | ICC (95% CI) |
| EATVI | 0.901 (0.766, 0.960) | 0.912 (0.795, 0.964) |
| LVGRS (%) | 0.852 (0.637, 0.941) | 0.825 (0.584, 0.936) |
| LVGCS (%) | 0.814 (0.593, 0.922) | 0.871 (0.703, 0.947) |
| LVGLS (%) | 0.878 (0.699, 0.951) | 0.848 (0.661, 0.936) |
| LVGRSDr (1/s) | 0.705 (0.396, 0.871) | 0.744 (0.467, 0.890) |
| LVGCSDr (1/s) | 0.685 0.354, 0.863) | 0.665 (0.320, 0.854) |
| LVGLSDr (1/s) | 0.854 (0.708, 0.947) | 0.880 (0.664, 0.954) |
| RVGRS (%) | 0.841 (0.646, 0.933) | 0.866 (0.698, 0.944) |
| RVGCS (%) | 0.854 (0.658, 0.940) | 0.893 (0.751, 0.956) |
| RVGLS (%) | 0.804 (0.568, 0.918) | 0.883 (0.730, 0.952) |
| RVGRSDr (1/s) | 0.647 (0.308, 0.842) | 0.668 (0.341, 0.853) |
| RVGCSDr (1/s) | 0.683 (0.135, 0.883) | 0.775 (0.055, 0.933) |
| RVGLSDr (1/s) | 0.754 (0.175, 0.917) | 0.719 (0.170, 0.900) |

Abbreviations: CI, confidence interval; ICC, intraclass correlation coefficient; EATVI, epicardial adipose tissue volume index; LVGRS, left ventricular global radial strain; LVGCS, left ventricular global circumferential strain; LVGLS, left ventricular global longitudinal strain; LVGRSDr, left ventricular global radial strain of diastolic rate; LVGCSDr, left ventricular global circumferential strain of diastolic rate; LVGLSDr, left ventricular global longitudinal strain of diastolic rate; RVGRS, right ventricular global radial strain; RVGCS, right ventricular global circumferential strain; RVGLS, right ventricular global longitudinal strain; RVGRSDr, right ventricular global radial strain of diastolic rate; RVGCSDr, right ventricular global circumferential strain of diastolic rate; RVGLSDr, right ventricular global longitudinal strain of diastolic rate.
